# Mode-Selective Raman Imaging of Metal–Organic Frameworks Reveals Surface Heterogeneities of Single HKUST‑1 Crystals

**DOI:** 10.1021/acsomega.5c00515

**Published:** 2025-07-25

**Authors:** Matheus Esteves Ferreira, Mariana Del Grande, Felipe Lopes Oliveira, Rodrigo Neumann Barros Ferreira, Ademir Ferreira da Silva, Pamela Costa Carvalho, Geisa Pires Nogueira de Lima, Ado Jorio, Mathias Steiner

**Affiliations:** † 376340 IBM Research, Rio de Janeiro 20031170, Brazil; ‡ Graduate Program of Technological Innovation – PPGIT, 28114 Universidade Federal de Minas Gerais (UFMG), Minas Gerais 31270901, Brazil; § Institute of Chemistry, 28125 Universidade Federal do Rio de Janeiro (UFRJ), Rio de Janeiro 21941909, Brazil; ∥ IBM Research, São Paulo 04007900, Brazil; ⊥ Department of Physics, Universidade Federal de Minas Gerais (UFMG), Minas Gerais 31270901, Brazil

## Abstract

Metal organic frameworks (MOFs) are nanoporous materials with high surface-to-volume ratios that have potential applications as gas sorbents. Sample quality is, however, often compromised and it is unclear how defects and surface contaminants affect the spectral properties of single MOF crystals. Raman microspectroscopy is a powerful tool for characterizing MOFs, yet spatial heterogeneity distributions of single MOF crystals have not been reported so far. In this work, we use Raman microspectroscopy to characterize spatially isolated, single crystals of the MOF species HKUST-1. In the first step, we validate the HKUST-1’s Raman spectrum based on DFT simulations, and we identify a previously unreported vibrational feature. In the second step, we acquire diffraction-limited, mode-selective Raman images of single HKUST-1 crystals that reveal how the spectral variations are distributed across the crystal surface. In the third step, we explore how multivariate data analysis can aid feature identification in Raman images of single MOF crystals. Finally, we statistically analyze the measured spectral peak positions and line widths for quantifying the variability occurring within the same crystal as well as between different crystals taken from the same batch. For enabling validation and reuse, we make the spectroscopic data and simulation code publicly available.

## Graphical abstract

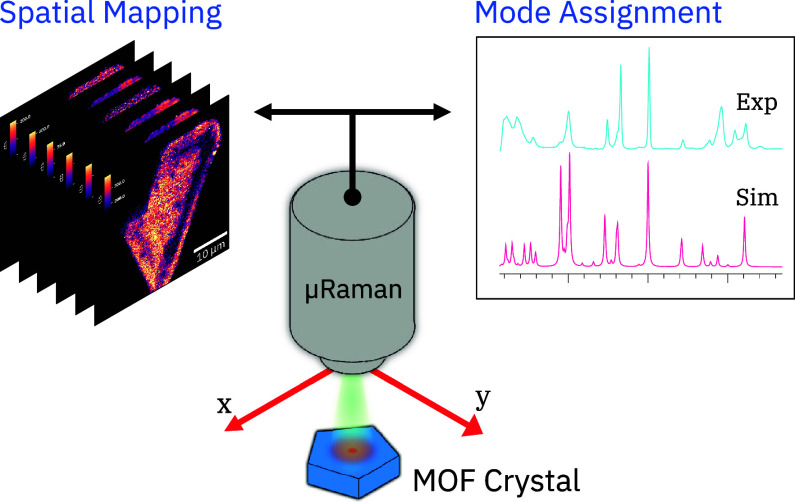

## Introduction

Metal organic frameworks (MOFs), a class of crystalline, nanoporous materials with high surface area and gas adsorption capacity are currently being researched for application in chemical separations.^1–3^ By coordinating metallic cluster and organic ligands with a broad range of framework topologies, a large variety of 3D structures can be obtained for supporting specific applications.^1,4^ However, the application performance of MOFs is impacted by their chemical quality at the molecular scale.

Raman microspectroscopy has been used to characterize MOF composition, structure, topology, as well as defects.^5,6^ In addition, the method was applied to MOFs for investigating molecular interactions,^7–11^ framework activation,^12,13^ degradation,^14^ and gas adsorption.^15^ Up to now, however, Raman studies of MOFs were performed mainly with bulk samples, with few studies exploring the use of Raman microscopic imaging.^6,15,16^

For determining how imperfections are distributed in single crystals and if the variations observed at the level of single crystals are representative of the bulk, it is necessary to first spatially isolate individual crystals and then microscopically resolve the spectroscopic signal at the crystal surface. By applying this approach to a number of crystals taken from the same batch, it is then possible to perform a statistical analysis of their spectroscopic properties. Based on this analysis, the sample heterogeneity can be quantified in terms of spectroscopic metrics, such as spectral shifts and line widths, that are characteristic of single crystals as well as of the bulk.

In this work, we apply micro-Raman spectroscopy to investigate the surface heterogeneity of MOFs in the specific case of HKUST-1. We evaluate its Raman spectrum by comparing measured and simulated data and by identifying the Raman bands that carry information about defects and contaminants. We microscopically resolve the surface of a single crystal and demonstrate how heterogeneity is reflected by mode-selective scattering intensities. We analyze the spectral peak positions and line widths and compare them with the results obtained from 100 spatially isolated single crystals for establishing a Raman spectrum that is representative of the batch. In the following, we briefly introduce the materials and methods used in our investigation.

## Materials and Methods

### Sample Preparation

HKUST-1 metal organic frameworks (copper benzene-1,3,5-tricarboxylate) were purchased as a powder (Basolite C300, Sigma-Aldrich). Raman samples were prepared by picking up small amounts of powder using a disposable pipet tip and spreading the material on top of a glass microscope slide.

### Micro-Raman Spectroscopy

The selection of single HKUST-1 crystals for Raman investigation was done by microscopically mapping an area of 4 mm × 4 mm on a glass microscope slide with a 10×/0.25 NA objective (EC-EPIPLAN, Zeiss) in a confocal microscope setup (Alpha 300 RAS, Witec). The resulting sample map was used to identify regions with spatially isolated single crystals.

Additional images taken with a 63×/0.75 NA objective (LD Plan-NEOFLUAR, Zeiss) were used to select crystals with diameters between 1 and 20 μm and to measure their respective center positions.

Raman spectra were acquired with a 100×/0.9 NA objective (EPIPLAN-Neofluar, Zeiss) in the spectral range of 70–3500 cm^–1^ using a λ = 532 nm solid-state laser (Witec) set to 1 mW. We used a 600 grooves/mm grating, blazed at 500 nm and centered at 2047.09 cm^–1^. The scattered light was detected with a EMCCD camera (Newton, Andor), Peltier-cooled to −59 °C, that has a horizontal resolution of 1600 pixels.

### Mode-Selective Raman Imaging

Mode-selective Raman images were taken on spatially isolated, single HKUST-1 crystals with a 100×/0.9 NA microscope objective (EPIPLAN-Neofluar, Zeiss) by raster-scanning a 40 μm × 40 μm sample area with a pixel side length of 200 nm. The integration time was set to 0.5 s.

### Spectral Data Analysis

A preprocessing step was carried out on the raw spectra using the Witec Project 6 software (Oxford Instruments). First, a cosmic ray reduction (CRR) algorithm was executed with filter size and dynamic factor set to 3 and 3, respectively. Second, a background removal step was carried out with the shape method, with a size of 110. Then, each spectrum *S*_*i*_ was normalized to the intensity of the spectral band at 1006 ± 25 cm^–1^. A nonlinear, least-squares curve fitting of Lorentzian peak functions was performed using the *LMFIT* python library^17^ for extracting the experimental peak positions, spectral line widths (fwhm), and relative band intensities. The standard deviation was clipped at the 99% percentile to limit the influence of cosmic rays on the quality of the spectra.

### Principal Component Analysis (PCA)

Principal Component Analysis (PCA) was performed using the *scikit-learn* python library.^18^ The PCA parametrization was carried out by defining a 6-dimensional space based on the single-crystal Raman maps. All data sets were transformed using the principal components of the single-crystal data set.

### Raman Simulations

All simulations were based on Density Functional Theory (DFT) under periodic boundary conditions, using the semilocal formulation of the Perdew–Burke–Ernzerhof (PBE) exchange-correlation functional^19^ in combination with the semiempirical correction for dispersive interactions D3 as proposed by Grimme using the Becke–Johnson damping function variation.^20,21^

The Kohn–Sham equations were solved using the Quickstep code from the CP2K v2023.1 package^22–24^ employing the Orbital Transformation (OT) for wave function optimization.^25,26^ Core electrons were treated with analytical pseudopotentials proposed by Goedecker, Teter, and Hutter (GTH).^27,28^ Valence electrons were expanded on a mixed basis of plane waves and Gaussian waves. The Gaussian basis used was a triple-ζ with two sets of polarization functions.^29^ For plane waves, a cutoff energy of 1200 Ry was used, mapped into a five-level grid with a relative cutoff energy of 50 Ry, with the Brillouin zone integration restricted to the Γ-point.

The atomic positions and cell parameters were derived from the *DOTSOV02*_*clean* crystallographic information file (CIF) part of the CoRE MOF 2014 data set.^30^ The structure was fully optimized until the total forces were below 1.0 millihartree/bohr (a mean square value below 0.7) and total pressure below 100 bar, using the Broyden–Fletcher–Goldfarb–Shanno (BFGS) minimization algorithm with memory limited to 25 steps (L-BFGS).

The vibrational modes and the intensity of the Raman spectra were both calculated based on the finite differences method under the harmonic approximation, where the potential energy surface was obtained by means of density functional theory using the CP2K v2023.1 ^22–24^ and phonopy^31,32^ packages. We employ a hybrid framework that combines Gaussian-type orbitals with plane-wave basis sets, balancing computational efficiency and precision, as implemented in CP2K.^24^ This method has been used to accurately predict representative condensed matter systems^23^ and it is aimed at massively parallel calculations, being especially suitable to treat extended materials such as MOFs. More details on the simulations can be found in the Supporting Information, which also includes links to the data and code repositories.

## Results and Discussion

### Single-Crystal Raman Spectrum of HKUST-1

The HKUST-1 sample studied in this investigation consists of octahedral crystals with diameters ranging from 1 to 20 μm. Figure 1a shows a brightfield microscopy image of a representative, spatially isolated HKUST-1 crystal. As an experimental reference, we plot in the inset of Figure 1a HKUST-1 Raman spectrum obtained at the center position of the crystal shown in Figure 1a. We will now identify the measured HKUST-1 Raman bands and assign the principal vibrational modes based on our simulation results.

**Figure 1:**
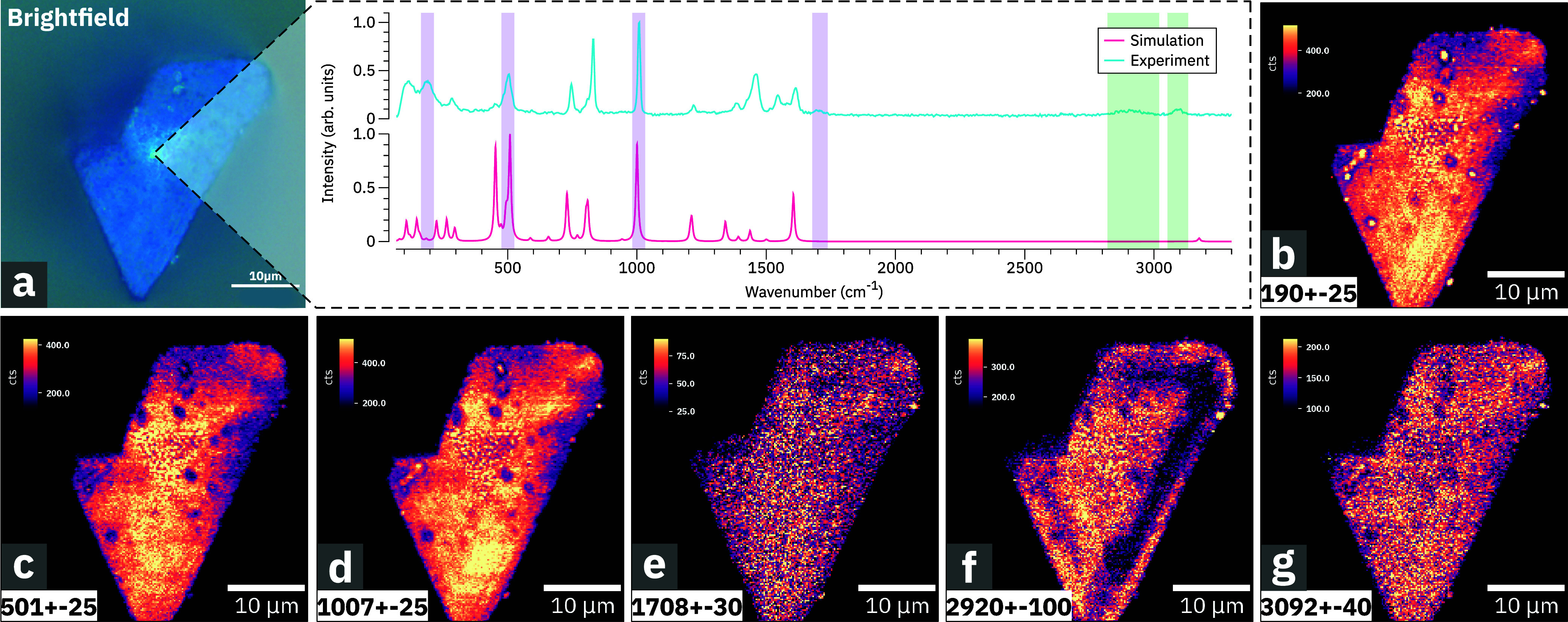
Mode-selective Raman imaging of a single, spatially isolated HKUST-1 crystal. (a) Brightfield microscopy image of a single HKUST-1 crystal. (b–g) Confocal Raman images of the same HKUST-1 crystal representing the integrated intensities of select Raman bands (values in the boxes are in cm^–1^) highlighted in the inset. The color-shaded areas (purple: previously reported; green: not reported) highlight the positions of the selected Raman bands in Tables 1 and 2. (Inset) Averaged Raman spectrum acquired at the center position of a spatially isolated single HKUST-1 crystal [blue] and simulated Raman spectrum [pink] for comparison.

In order to perform the Raman band assignment, we simulated the vibrational modes using density functional theory; see Methods section. A comparison of measured and simulated spectra is shown in the inset of Figure 1. For each mode, we have calculated mode symmetry, center frequency, and the respective vector representation along with the scattering intensities for both parallel and perpendicular incidence. Overall, the simulated spectrum in the inset of Figure 1 is composed of 468 independent, vibrational modes and their properties are provided in the Supporting Information.^33^

For the purpose of the analysis of surface heterogeneities at the single-crystal level, we focus our investigation on a set of Raman modes that carry information about structural defects and impurities. The selected modes are summarized in Tables 1 and 2 and in Figure 2 we show their vector representations.

**Figure 2:**
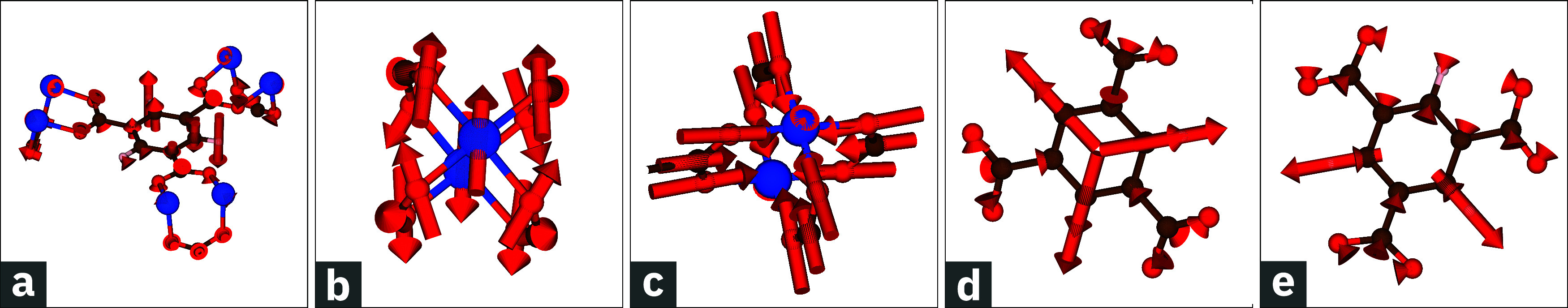
Vibrational modes of HKUST-1. (a–e) The vectors indicate select vibrations summarized in Tables 1 and 2 giving rise to the measured Raman bands at (a, b) 174–283, (c) 500 (d) 1007, and (e) 3090 cm^–1^, respectively. The respective spectral segments are highlighted in the inset of Figure 1 by purple shadows that are overlaid with Raman spectra. Copper, oxygen and carbon atoms are represented by blue, red, brown colors, respectively.

**Table 1:** Vibrational Assignment of Previously Reported HKUST-1 Modes^a^

center (cm^–1^)	Δ center (cm^–1^)	simulated mode center (cm^–1^)	symmetry	mode description	ref
7.96	1.15	185.07	A_1g_	τ_op_(CH)_b_/δ(OCuO)	^5^
500.02	1.13	508.75	A_1g_	ν_ip_(OCuO)	^5^
1007.00	0.93	1000.53	A_1g_	in-phase breathing of the benzene ring	^5^
1705.17	8.84			ν_op_(COOH)	^5^

a Mode Assignment of Select Raman Bands. ν = Stretching; δ = Bending; w = Wagging; ip = In Phase; op = Out of Phase; τ = Twisting; b = Trimesic Group.

**Table 2:** Vibrational Assignment of High-Frequency HKUST-1 Modes^a^

center (cm^–1^)	Δ center (cm^–1^)	simulated mode center (cm^–1^)	symmetry	mode description
2918.20	7.53			
3090.84	3.55	3175.11	A_1g_	ν_op_(CH)_b_

a Mode Assignment of Select Raman Bands. ν = Stretching; δ = Bending; w = Wagging; ip = In Phase; op = Out of Phase; τ = Twisting; b = Trimesic Group.

The modes below 500 cm^–1^ are mainly associated with vibrations related to either Cu-O or Cu-Cu bonds.^5^ In Figure 2a,b, we show both the representation of the torsion of the aromatic ring of the trimesic group and the bending mode of the *O-Cu-O* group, respectively. In addition, Figure 2c,d visualize the symmetric stretch vibrations of the *Cu-O* bonds as well as the in-phase breathing vibration of the aromatic ring.

Two measured Raman bands centered at 1705 and 2918 cm^–1^, see Figure 1, do not appear in the simulated spectra. The band at 1705 cm^–1^ was assigned to the out-of-phase bending mode of COOH groups and correlated with local defects of the framework structure originating from carboxyl groups.^5^

To the best of our knowledge, the spectral features at 2918 and 3090 cm^–1^ have not been previously reported. Based on our simulation results, we assign the band at 3090 cm^–1^ to the out-of-phase CH stretch of the aromatic ring in the trimesic group of HKUST-1. The visualization of this vibration is shown in Figure 2e.

By comparing our simulation results, we conclude that the band at 2918 cm^–1^ cannot be linked to the HKUST-1 crystal itself. The feature rather attests to presence of surface contaminants. To confirm this conclusion, we have performed spectroscopic measurements with a reference HKUST-1 sample which was synthesized independently. The reference sample exhibits the Raman band at 3090 cm^–1^, however, it lacks the feature centered at 2900 cm^–1^, see Supporting Information. However, confirming the chemical identity of the adsorbant species would require further research beyond the scope of this paper.

### Mode-Selective Raman Imaging of Single HKUST-1 Crystals

Having established the Raman spectrum of HKUST-1 at the single-crystal level, we now investigate if surface heterogeneities emerge at the surface of a single HKUST-1 crystal. By raster-scanning the surface of a spatially isolated HKUST-1 crystal with a confocal laser microscope, we have acquired a series of Raman images, see Figure 1b–g, for select spectral bands that correspond to the modes summarized in Tables 1 and 2. To ensure stable focus conditions at the surface of the crystal and for maintaining an acceptable signal-to-noise ratio (SNR), we have chosen a crystal that was oriented parallel to the focal plane. Previous studies have identified the surface facet of octahedral HKUST-1 crystals as the [111] plane.^100^ As the Raman signal contains information about specific structural defects and impurities, the images reveal mode-selective heterogeneity at the surface of the same crystal.

Specifically, in Figure 1b, which maps the scattering intensities related to the δ_op_(O-Cu-O) vibration spectrally centered at 190 cm^–1^, we observe localized centers with enhanced scattering intensities. The observed enhancement of Raman scattering intensity could be related to the presence of metal defects localized close to the crystal surface^34,35^ that cause local resonance enhancements^36^ or heating effects.

In contrast, Figure 1c,d, which correspond to bands centered at 501 and 1007 cm^–1^, show reduced scattering intensities at the same locations, leading to contrast inversion with dark centers at the location of the defects. However, all three images Figure 1b–d show the same, spatially extended heterogeneity at the bottom vertex of the crystal that could be due to changes in surface scattering density. In Figure S1, we provide the normalized spatial maps for all bands of HKUST-1.

The image in Figure 1e, spectrally located at 1708 cm^–1^, shows a rather homogeneous spatial intensity distribution with low scattering intensities, attesting to the absence of carboxyl group-related defects at the crystal surface.^5^

However, the image in Figure 1f for the Raman band spectrally centered at 2920 cm^–1^ shows a large, connected area at the right-hand side of the crystal surface where the scattering intensity is significantly reduced. Following the spectral assignment in the previous subsection, we conclude that this signal attests to the presence of contaminants at the surface of the crystal.

We note that the image for the newly assigned spectral band at 3092 cm^–1^, which is due to the out-of-phase CH stretch of the benzene ring of the trimesic group, ν_op_(CH), is mostly homogeneous across the surface, with the exception of some localized defects that are consistent with the other images.

### Principal Component Analysis for Identifying Single-Crystal Image Features

After having established that Raman imaging can be used to map surface heterogeneity in single HKUST-crystals, see Figure 1, we now explore if multivariate data analysis can be used to reveal spatial features that are not visible in the mode-selective Raman images.

Principal component analysis (PCA) allows the transformation of higher-dimensional spectroscopic data into a lower-dimensional space and captures features within a select number of principal components.^37,38^ In the present case, the Raman image data shown in Figure 1 were normalized and projected onto 6 principal components whose spectral distributions are plotted in Figure 3a. The spectral distribution of the first principal component, PC1, resembles the original HKUST-1 spectrum, while PC2 is mainly a negative image of PC1. PC3 to PC6 have both positive and negative spectral contributions. PC2 has negative peak amplitudes across the spectral range and exhibits no significant spatial variability along the crystal edge. Therefore, we conclude that heterogeneities along the edge are of topographical origin, rather than chemical.

**Figure 3:**
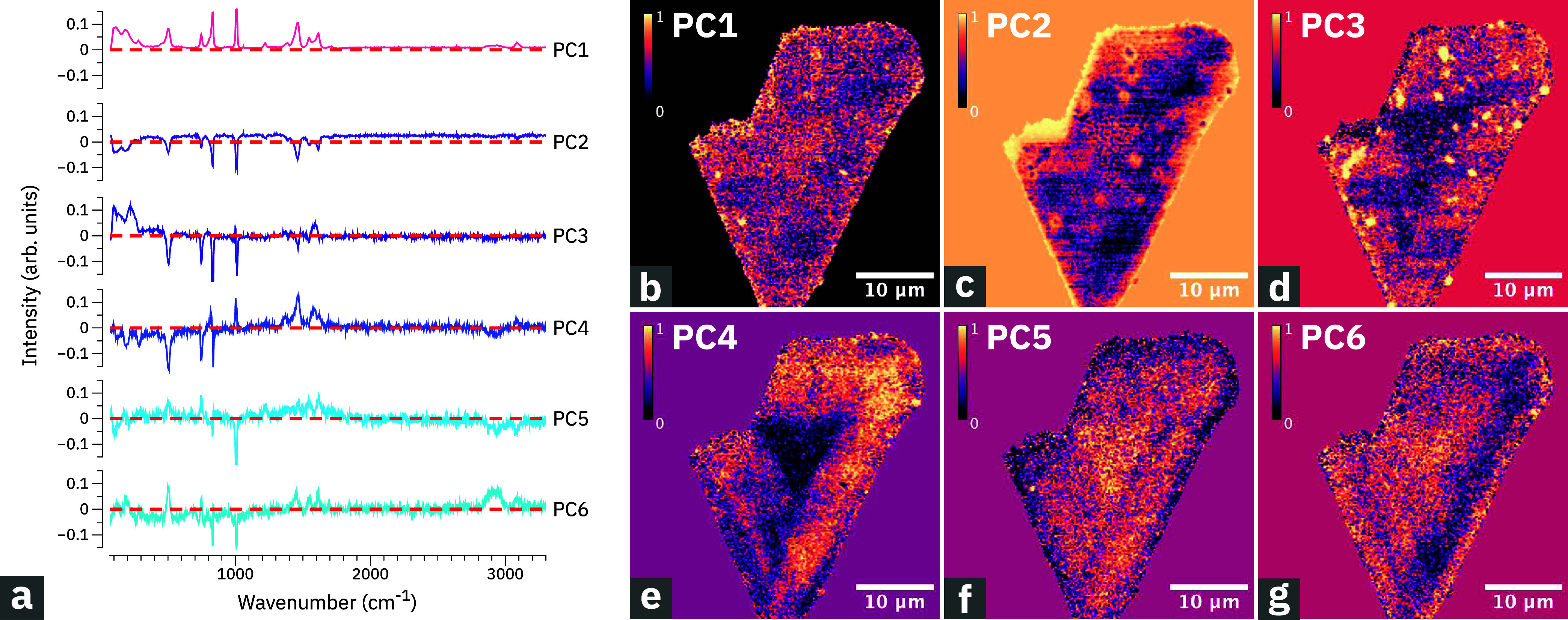
Principal component analysis of HKUST-1 single-crystal Raman data. (a) Spectral distribution of the six principal components (PC1–PC6). The dashed red line represents PC = 0. (b–g) Images with spatial distribution of principal components 1–6 for the same HKUST-1 crystal.

Component-selective Raman images were created by computing a 6-dimensional principal component space pixel-wise from the Raman spectral data. The dimensionality transformation operation yields a 6-channel data-cube where each channel represents one principal component, represented in Figure 3b–g. The images created for principle components 1 and 2, respectively, mainly exhibit contrast variations at the position of defects that are consistent with the mode-selective Raman images of Figure 1. However, the images for principle components 3–6 reveal a triangular area, having a side length of about 10 μm, which is located at the center of the crystal. This area was not readily visible in any of the mode-selective Raman images of Figure 1, although it is discernible in the normalized maps shown in Figure S1.

In the images representing principle components 3 and 4, respectively, the triangular area exhibits negative image contrast, while the image contrast is positive in the case of principle components 5 and 6. By looking at the spectral distributions of the principal components in Figure 3a, this could indicate a relative increase of spectral weight in the range between 500 and 750 cm^–1^ and a decrease of spectral weight in the region between 800 and 1000 cm^–1^.

### Spectral Variability of Single-Crystal HKUST-1

Based on the experimental findings that single crystals of HKUST-1 show spatial variations of Raman scattering intensities across their surface, see Figure 1, the question is how the occurring spectral variations can be quantified. Potentially useful metrics for variability analysis are the spectral peak positions and line widths, respectively, of the measured Raman bands. The experimental values can ultimately serve as a reference for spectroscopic sample-to-sample comparisons.

For analyzing spectral variations within the same crystal, we measured the Raman spectra of a spatially isolated, single HKUST-1 crystal at 100 different positions distributed across the crystal surface. We have statistically evaluated the average spectral peak positions as well as the spectral line widths, along with their respective standard deviations. The data were obtained by fitting Lorentzian line shape functions to the experimental data; see the Methods section.

In Figure 4a, we plot the spectrum in solid colors and the standard deviation in shaded colors. In the low-frequency regime below 400 cm^–1^, as well as in the spectral ranges between 1100 and 1800 cm^–1^ and 2800–3100 cm^–1^, we observe high spectral variability. Below 400 cm^–1^, most modes are related to Cu­(II) open metal sites and the Raman peak positions are subject to shifts caused by the presence of water molecules^9,12,39^ which are sensitive to local heating induced by the laser illumination. The increased variability in the spectral range between 1100 and 1800 cm^–1^ could also be due to local heating. The band at 2920 cm^–1^, which was assigned to surface contaminants or adsorbants, also shows higher variability in the present case. Note that this signal was absent in some reference crystals taken from the same batch.

**Figure 4:**
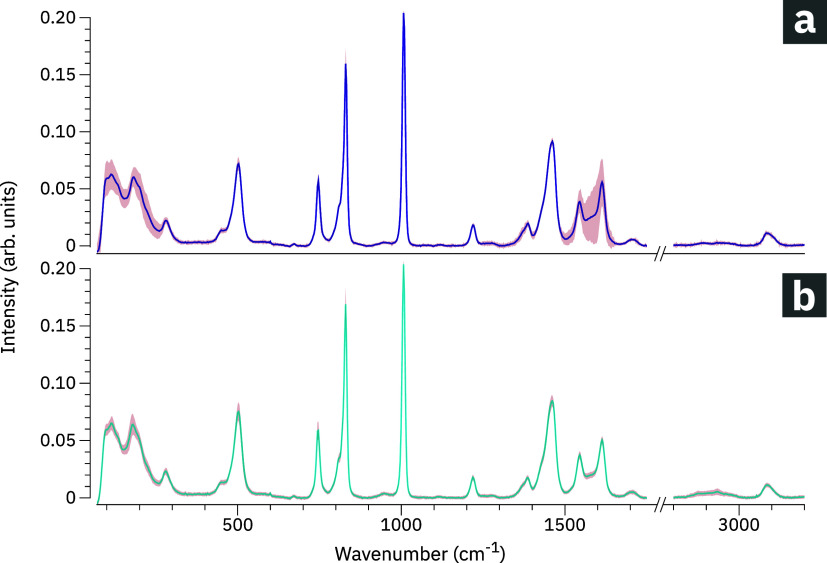
Spectral variability of single HKUST-1 crystals. (a) Raman spectrum obtained by averaging 100 spectra obtained at different positions of the same single crystal. (b) Raman spectrum obtained by averaging 100 spectra recorded at the center positions of 100 different single crystals of the same batch. Shaded areas: standard deviation.

In contrary, the bands between 400 and 1100 cm^–1^ which are either related to the vibration of the benzene rings or to the OCO vibrations show little spectral variability. The peak position of the Raman band at 1007 cm^–1^, which represents the in-phase breathing vibration of the aromatic rings, shows the lowest variability in our analysis. Due to its high robustness and stability, we have chosen it as a spectral normalization reference to allow for analysis of crystal-to-crystal variations. The spectral peak positions and line widths, along with their respective standard deviations, are listed in Table 3.

**Table 3:** Experimental Raman Spectral Peak Positions and Line Widths, Respectively, of HKUST-1^a^

peak center (cm^–1^)	Δ center (cm^–1^)	fwhm (cm^–1^)	Δ fwhm (cm^–1^)	rel. height (arb. units)
95.0	0.6	13.6	2.4	0.2
111.8	1.2	23.2	7.6	0.2
132.5	3.1	33.8	10.5	0.2
178.5	2.9	43.0	11.0	0.2
204.1	8.4	52.6	12.1	0.1
280.8	1.1	21.3	3.9	0.1
456.0	7.9	30.7	15.2	0.0
500.9	0.3	24.3	1.0	0.3
745.6	0.3	11.4	1.0	0.3
814.0	1.6	22.2	4.2	0.1
829.5	0.1	8.9	0.5	0.8
1006.9	0.2	9.0	0.5	1.0
1219.0	0.2	15.4	0.5	0.1
1275.6	2.2	21.5	6.7	0.0
1386.0	2.3	13.4	7.8	0.1
1447.8	1.9	31.9	2.8	0.2
1463.3	0.6	17.0	2.2	0.3
1547.0	0.7	26.2	2.1	0.2
1610.9	0.4	25.5	1.4	0.2
2915.0	1.2	142.9	8.3	0.0
3089.6	0.3	37.3	1.3	0.1

a The Spectral Variability is Provided as the Standard Deviation. The Data Represents 100 Single-Shot Raman Spectra Taken at the Center Positions of 100 Different Single Crystals Taken from the Same Sample Batch.

For quantifying crystal-to-crystal variability occurring in the same sample batch, we acquired Raman spectra at the center position of 100 spatially isolated single HKUST-1 crystals. The averaged spectrum, see Figure 4b, shows noticeable variability in the low-frequency regime below 400 cm^–1^, as well as in the spectral ranges between 1100 and 1800 cm^–1^ and 2800–3100 cm^–1^. This is in agreement with the spectral variability we observed on the single-crystal level.

As a key result of our study, the peak positions of Raman bands in the averaged spectra acquired (a) at 100 different positions of the same, single crystal and (b) at the center positions of 100 different, single crystals are consistent. However, spectral standard deviations are larger in case (a) than in case (b). This demonstrates that heterogeneity can be high within the same crystal, underscoring the relevance of spatially resolved investigations.

In Table 3, we summarize the spectral peak positions and line widths along with their respective standard deviations. The data can serve as a reference for lab characterization of HKUST-1 samples.

## Summary and Conclusions

In summary, we performed an analysis of mode-selective Raman scattering in single HKUST-1 crystals. Based on ab initio simulations, we have validated the principle Raman features of HKUST-1 and identified one Raman feature not previously reported elsewhere. We have microscopically resolved surface heterogeneities of spatially isolated single HKUST-1 crystals that are caused by local defects and surface contamination.

By analyzing Raman spectra acquired with the same single crystal as well as with a set of different crystals, we establish a representative, single-crystal HKUST-1 Raman spectrum. The spectral peak positions and line widths, along with their standard deviations, can serve as a reference for monitoring the MOF quality in the lab. The comparison of both sets of spectra reveals that spectral variability within the same crystal can be higher than that across a set of different crystals taken from the same batch.

Finally, we explored how principal component analysis can be applied for revealing features that cannot be straightforwardly identified in mode-selective Raman imaging. The analysis of the chemical origins of these features requires further research.

For studying the HKUST-1 surface with higher spatial resolution, at the level of single defects, we suggest to evaluate both tip-enhanced Raman spectroscopy^40,41^ as well as tip-enhanced infrared spectroscopy^42^ on the same sample. This allows for obtaining complementary, spatial and spectral information that is not available with diffraction-limited Raman microspectroscopy method applied here.

To allow for reuse and validation of our results, we have made the experimental data and simulation code available in public repositories.

## Supplementary Material



## Data Availability

HKUST-1 samples can be provided for complementary analysis on reasonable request. The experimental and simulation data can be found in the following repository: Zeonodo: https://zenodo.org/uploads/14165785. The code for the simulation of the Raman spectra of MOFs is provided in GitHub: https://github.com/neumannrf/electronic-structure-experiment.

## Notes

Powder X-ray diffraction data (XRD spectra) of the samples analyzed in this study are provided; further information on the Raman simulation methods and the mode-selective Raman maps are provided (PDF)
