# Supplementary material for: Mode-Selective Raman Imaging of Metal–Organic Frameworks Reveals Surface Heterogeneities of Single HKUST‑1 Crystals
Source: ACS Omega. 2025 Jul 25;10(30):33393–400. doi: 10.1021/acsomega.5c00515 (PMC12332789; doi:10.1021/acsomega.5c00515)
Supplement: Supplementary file 1 [file ao5c00515_si_001.pdf]

Mode-selective Raman imaging of metal-organic frameworks reveals surface heterogeneities of single HKUST-1 crystals

Supporting Information

Matheus Esteves Ferreira,[†,‡] Mariana Del Grande,[†] Felipe Lopes Oliveira,[¶] Rodrigo Neumann Barros Ferreira,[†] Ademir Ferreira da Silva,[†] Pamela Costa Carvalho,[§] Geisa Pires Nogueira de Lima,[†] Ado Jorio,[‡,∥] and Mathias Steiner[*,†]

†*IBM Research, Rio de Janeiro, Brazil, 20031170*

‡*Graduate Program of Technological Innovation - PPGIT, Universidade Federal de Minas Gerais (UFMG), Minas Gerais, Brazil, 31270901*

¶*Institute of Chemistry, Universidade Federal do Rio de Janeiro (UFRJ), Rio de Janeiro, Brazil, 21941909*

§*IBM Research, São Paulo, Brazil, 04007900*

∥*Department of Physics, Universidade Federal de Minas Gerais (UFMG), Minas Gerais, Brazil, 31270901*

E-mail: mathiast@br.ibm.com

Raman Simulations

The vibrational modes were calculated under the harmonic approximation, where the potential energy surface (U) obtained by density functional theory is expanded in a Taylor series

$$U(q_k) = U_0 + \sum_k \left(\frac{\partial U}{\partial \vec{u}_k}\right) \vec{u}_k + \frac{1}{2}\sum_{j,k} \vec{u}_j \left(\frac{\partial^2 U}{\partial \vec{u}_j \partial \vec{u}_k}\right) \vec{u}_k + ... \qquad \text{(eq. S1)}$$

where U_0 is the ground state energy of the stable geometry, $\vec{u}_k$ is the cartesian coordinate displacement of the k_{th} atom, $\frac{\partial U}{\partial \vec{u}_k}$ is the gradient of the electronic energy along this direction, and $H_{j,k} = \frac{\partial^2 U}{\partial \vec{u}_j \partial \vec{u}_k}$ is the second derivative (or Hessian matrix) along these directions. When the stable geometry correspond to a minimum on the potential surface the gradient terms vanish.

Phonons were computed by diagonalizing the dynamical matrix obtained from the variation of atomic forces due to finite atomic displacements of 0.005 Å. The diagonalization process was carried out using the phonopy package.[1, 2]

For a Stokes process, the differential scattering cross section of the *m-th* vibrational mode is given by[3]

$$\frac{d\sigma}{d\Omega} \propto \frac{(\omega_0 - \omega_m)^4}{\omega_m} |\mathbf{e}_i \cdot \alpha^m \cdot \mathbf{e}_s|^2 (n_m + 1) \qquad \text{(eq. S2)}$$

where $\mathbf{e}_i$ and $\mathbf{e}_s$ are the unit polarization vectors of incident and scattered radiation, ω_0 is the frequency of incident radiation, ω_m is the frequency of the phonon mode, n_m is the Bose-Einstein distribution given by $n(\omega_m) = 1/(1 - e^{-\hbar\omega_m/k_B T})$ and α^m is the Raman tensor given by

$$\alpha^m_{ij} = \sqrt{V} \sum_{\alpha,\beta} \frac{\partial \chi_{ij}}{\partial r_{\alpha,\beta}} \tau^m_{\alpha,\beta} \qquad \text{(eq. S3)}$$

where V is the unit cell volume, $r_{\alpha,\beta}$ is the α coordinate of β-th atom in the unit cell,

and $\tau^m_{\alpha,\beta}$ corresponds to the normal vector of the m-th vibrational mode on the β atom.

The linear dielectric susceptibility tensor, χ_{ij} is calculated for each distorted structure using the linear response method as implemented in CP2K.

To simplify the dependence on the polarization vectors $\mathbf{e}_i$ and $\mathbf{e}_s$ the Raman invariants (a, δ, and γ) can be defined based on the Placzek approximation, in terms of the Raman tensor α, with $I^m_{tot} = |\mathbf{e}_i \cdot \alpha^m \cdot \mathbf{e}_s|^2$. The set of invariants consists of the mean polarizability a, the antisymetric anisotropy δ and the anisotropy γ, given by:

$$a^2 = \frac{1}{9}\left[|\alpha_{xx} + \alpha_{yy} + \alpha_{zz}|^2\right] \quad \text{(eq. S4)}$$

$$\delta^2 = \frac{3}{4}\left[|\alpha_{xy} - \alpha_{yx}|^2 + |\alpha_{xz} - \alpha_{zx}|^2 + |\alpha_{yz} - \alpha_{zy}|^2\right] \quad \text{(eq. S5)}$$

$$\begin{aligned}\gamma^2 = &\frac{1}{2}\left[|\alpha_{xx} - \alpha_{yy}|^2 + |\alpha_{xx} - \alpha_{zz}|^2 + |\alpha_{yy} - \alpha_{zz}|^2\right] \\ &+\frac{3}{4}\left[|\alpha_{xy} + \alpha_{yx}|^2 + |\alpha_{zx} + \alpha_{xz}|^2 + |\alpha_{yz} + \alpha_{zy}|^2\right]\end{aligned} \quad \text{(eq. S6)}$$

Thus, the Raman intensity with parallel ($I^m_{\parallel}$) and perpendicular ($I^m_{\perp}$) polarization for the *m-th* mode can be calculated as

$$I^m_{\parallel} = \frac{45a^2 + 4\gamma^2}{45} \quad \text{(eq. S7)}$$

$$I^m_{\perp} = \frac{3\gamma^2 + 5\delta^2}{45} \quad \text{(eq. S8)}$$

and thus

$$I^m_{tot} = I^m_{\parallel} + I^m_{\perp} \quad \text{(eq. S9)}$$

To generate the simulated spectra the Raman intensities were convoluted with a Lorentzian curve of width $\gamma = 5.0$ cm^{-1} given by

$$f(\omega) = \frac{1}{\pi}\left[\frac{\gamma}{(\omega - \omega^n)^2 + \gamma^2}\right] \cdot I^n \qquad \text{(eq. S10)}$$

where ω^n is the position of the n-th Raman peak, I^n is the intensity of the n-th Raman peak and γ is the width of the peak.

Mode-selective Raman images of single HKUST-1 crystal

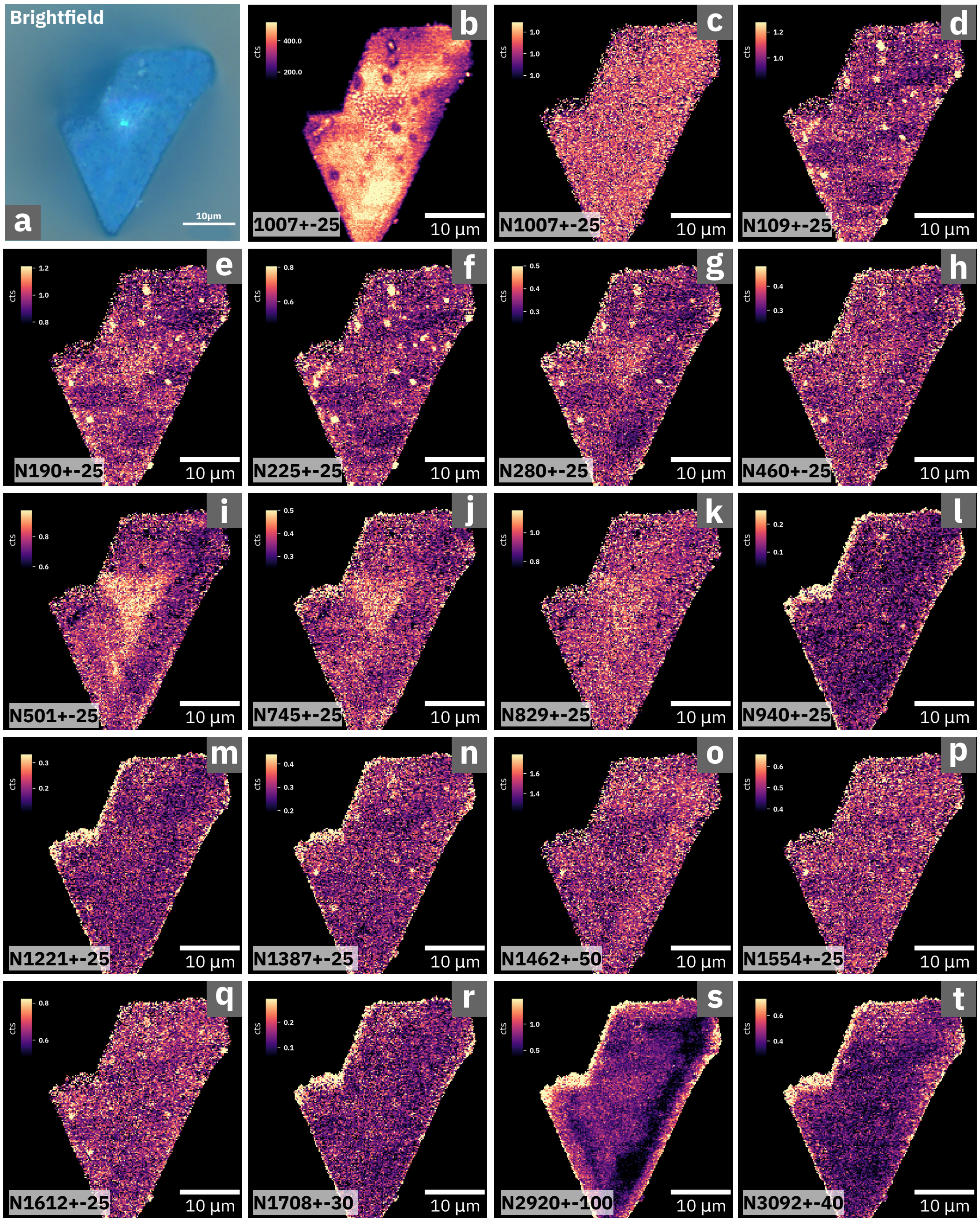

Figure S1: Normalized Mode-selective Raman imaging of a single, spatially isolated HKUST-1 crystal(a) Brightfield microscopy image of a single HKUST-1 crystal. (b) 1007 ± 25 rel. cm^{-1} reference band. (c-t) Confocal Raman images of the same HKUST-1 crystal representing the normalized integrated intensities of select Raman bands in relation to the reference band

Powder X-Ray of Basolite C300

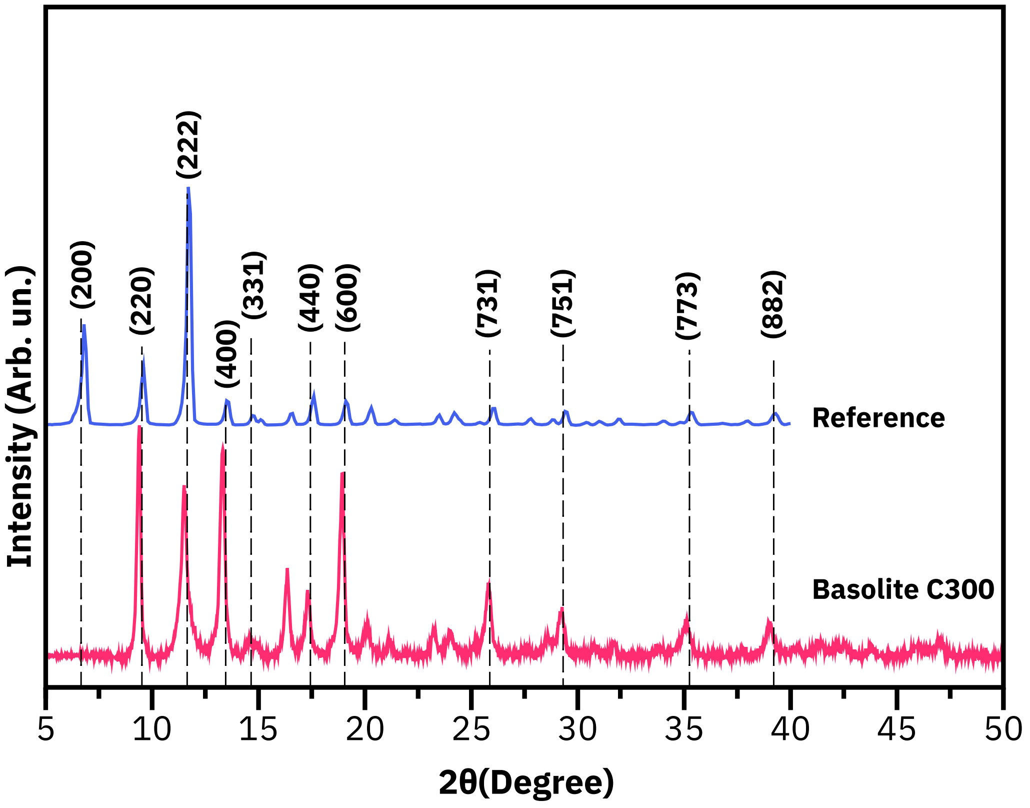

Figure S2: Powder X-Ray driffraction of HKUST-1 samples. Labels represent the miller indices of the respective peaks according to reference [4]

[1] Atsushi Togo. "First-principles Phonon Calculations with Phonopy and Phono3py". en. In: *Journal of the Physical Society of Japan* 92.1 (2023), p. 012001. DOI: 10.7566/JPSJ.92.012001.

[2] Atsushi Togo et al. "Implementation strategies in phonopy and phono3py". en. In: *Journal of Physics: Condensed Matter* 35.35 (2023), p. 353001. DOI: 10.1088/1361-648X/acd831.

[3] Derek A. Long. *The Raman Effect: A Unified Treatment of the Theory of Raman Scattering by Molecules*. en. 1st ed. Wiley, 2002. ISBN: 978-0-471-49028-9 978-0-470-84576-9. DOI: 10.1002/0470845767.

[4] *ACS Material Metal-Organic Framework Cu-BTC*. Tech. rep. ACS Material.
